# Bioinputs from *Eugenia dysenterica* DC. (Myrtaceae): Optimization of Ultrasound-Assisted Extraction and Assessment of Antioxidant, Antimicrobial, and Antibiofilm Activities

**DOI:** 10.3390/molecules30051115

**Published:** 2025-02-28

**Authors:** Fernando Gomes Barbosa, Gabriel Fernandes Silva, Valter Lúcio Pereira de Oliveira, Lorrainy Alves Cassemiro Kubijan, Leonardo Gomes Costa, Anielly Monteiro de Melo, Monatha Nayara Guimarães Teófilo, Cristiane Maria Ascari Morgado, André José de Campos, Josana de Castro Peixoto, Leonardo Luiz Borges, Carlos de Melo e Silva Neto, Eliete Souza Santana, Joelma Abadia Marciano de Paula

**Affiliations:** 1 Laboratory for Research, Development and Innovation of Biodiversity Products, State University of Goiás, Câmpus Central, Anapolis 75132-903, GO, Brazillorrainy@aluno.ueg.br (L.A.C.K.);; 2 Postharvest Laboratory, State University of Goiás, Câmpus Central, Anapolis 75132-903, GO, Brazil; 3 Evangelical University of Goiás, Anapolis 75083-515, GO, Brazil; 4 Federal Institute of Goiás, Innovation Pole, Goiânia 74968-755, GO, Brazil; 5 Laboratory of Microbiology, State University of Goiás, Câmpus Central, Anapolis 75132-903, GO, Brazil

**Keywords:** bioinputs, cagaita, cerrado, phenolic compounds, response surface methodology

## Abstract

By-products of fruit processing may contain bioactive compounds with potential application as bioinputs. This study optimized the ultrasound-assisted extraction (UAE) of phenolic compounds from the by-products of *Eugenia dysenterica* DC (Myrtaceae) fruit to obtain bioinputs with antioxidant, antimicrobial, and antibiofilm activities. The fruit by-products (seeds and peels) were subjected to UAE optimization using the Box–Behnken design and response surface methodology. Two optimal conditions were defined: 1-plant material/solvent ratio of 0.01 g/mL, time of 40 min, and ethanol content of 30%; 2–0.19 g/mL, 39 min, and 46%. The bioinputs (liquid extract—LQE; lyophilized extract—LYE), obtained under the optimal conditions, were tested for antioxidant activity (ABTS, FRAP, and DPPH). LQE: 1633.13 µM Trolox/g, 1633.60 µM FeSO_4_/g and 73.35 g sample/g DPPH; LYE: 1379.75 µM Trolox/g, 1692.09 µM FeSO_4_/g and 83.35 g sample/g DPPH. For antimicrobial activity, both extracts presented MBC < 62.5 mg/mL and MIC and MBC of 2.5 mg/mL for *P. aeruginosa*. LQE presented antibiofilm action for *S. coagulase* (50 mg/mL) and *Streptococcus* spp. (12.5 mg/mL); LYE for *P. aeruginosa* (50 mg/mL; 12.5 mg/mL), *E. coli* (25 mg/mL). The bioinputs obtained by UAE under optimized conditions for phenolic compounds present antioxidant, antimicrobial, and antibiofilm activities.

## 1. Introduction

Bioinputs are emerging as sustainable and strategic alternatives for modern agriculture, as they promote practices that combine productivity and environmental preservation. Defined as products of biological origin used in agricultural and livestock management, these inputs include microorganisms, plant extracts, and biofertilizers, emphasizing their ability to reduce dependence on synthetic chemicals and mitigate environmental impacts [1]. In Brazil, the National Bioinputs Program consolidates public policies aimed at stimulating the production and utilization of these inputs, including the use of plant by-products [1,2]. Agroindustrial by-products are secondary materials generated in the industrialization process of agricultural products. When they have market potential, they are defined as co-products; when they do not yet have commercial value, they are waste [3].

Bioinputs offer opportunities for phytosanitary control and represent a sustainable innovation for agriculture. According to data from the Brazilian Agricultural Research Corporation (Embrapa), in Brazil, as of January 2025, there are records of five bioinputs that control the development of bacteria and fungi of commercial interest to agriculture [4].

In addition to their application in pest control and agricultural management, bioinputs have significant potential in disease treatment in the livestock sector, such as bovine mastitis, and in sanitary control in poultry farming. Characterized by inflammation of the mammary gland, bovine mastitis generally results from bacterial infections and can be influenced by several factors, such as inadequate management, housing conditions, and milking practices in dairy cattle [5]. The economic losses associated with mastitis, both in its clinical and subclinical manifestations, include treatment costs, milk disposal, temporary decrease in production, early slaughter, and mortality [6]. For poultry farming, the lack of sanitary conditions in any part of the process can favor microbial growth, causing economic and public health losses [7,8].

Previous studies on essential oils [9] and alcoholic extracts of aromatic plants [10] evaluated the antimicrobial effect on different pathogens that cause bovine mastitis, demonstrating that plant-based raw materials have the potential to control different strains of microorganisms that cause the disease.

Among the natural alternatives for controlling microorganisms, bioactive compounds extracted from plants of the family Myrtaceae have been extensively studied due to their antimicrobial potential and the presence of phenolic compounds [11,12,13,14]. *Eugenia dysenterica* DC., popularly known as “cagaita”, is a fruit tree species native to the Cerrado (savanna biome) belonging to the family Myrtaceae, with wide distribution in Brazil. This fruit, with an acidic flavor and characteristic aroma, is known for its importance in regional cuisine and traditional use in folk medicine, in addition to having potential use in the pharmaceutical industry due to its bioactive properties [15].

Vitamin C, folates, carotenoids, terpenes, as well as phenolic compounds and polyphenols, such as flavonoids (catechin, quercetin, epicatechin) and tannins (ellagitannins and proanthocyanidins) can be found in *E. dysenterica* [16,17]. In cooking, *E. dysenterica* fruit is used to make fermented beverages, juices, sweets, and jellies [18,19]. Studies indicate trypanocidal, anticholinesterase, antidiarrheal, anthelmintic, antileukemic, antimicrobial, and antioxidant action of the leaf and fruit extracts of *E. dysenterica* [18,20,21,22,23,24,25]. Other effects, such as hypoglycemic, neuroprotective, and gastroprotective, have also been attributed to *E. dysenterica* extracts [26,27,28]. All this potential is associated with the compounds produced by the species in response to internal or external stimuli [29].

Extraction techniques appropriate to the plant material and the classes of compounds of interest are essential to ensure the efficacy and effectiveness of plant extracts. Extraction techniques such as infusion and decoction are widely used in popular culture, but they do not take into account the quantification of bioactive compounds present in the preparation [30].

Ultrasound-assisted extraction (UAE) is used to optimize the extraction of bioactive compounds from plants. In this technique, mechanical waves are propagated in compression and rarefaction cycles, promoting the phenomenon called cavitation. This phenomenon causes a punctual increase in temperature and pressure in the medium through which the waves propagate, as well as the formation of microjets. The microjets promote the rupture of solid particles in the medium, optimizing the mass transfer process during extraction [31].

Variables such as extraction time, solvent type and concentration, and proportion of plant material are factors that influence the efficiency of UAE [32] of various compounds. Statistical optimization tools are valuable instruments to indicate the best extraction conditions for bioactive compounds. The Box–Behnken factorial design combined with the response surface methodology (RSM) allows the efficient exploration of several experimental variables, such as time, temperature, and solvent, aiming to maximize the yield and quality of the extracts obtained. Studies demonstrate the effectiveness of the combination of these two methods in obtaining plant extracts [32,33].

Previous studies have optimized the extraction of bioactive compounds from Myrtaceae species [34,35,36], but no research has focused on *Eugenia dysenterica* by-products as sources of bioinputs, especially using UAE. Compared to traditional extraction methods such as maceration or Soxhlet extraction, UAE offers increased efficiency, reduced solvent consumption, and enhanced compound preservation, making it a more sustainable approach [37,38,39]. In addition, UAE is an efficient, fast, and environmentally friendly method that can be scaled for industrial applications, standing out as an innovative technique to produce bioinputs on a large scale [40,41].

The present study aimed to optimize the UAE of phenolic compounds from the by-products of *Eugenia dysenterica* DC fruit in order to obtain bioinputs (liquid extract—LQE; lyophilized extract—LYE) and to evaluate their antioxidant, antimicrobial, and antibiofilm activities in bacteria isolated from broiler chickens, ATCC strains, and bovine mastitis.

## 2. Results and Discussion

### 2.1. Optimization Experiment for the Extraction of Phenolic Compounds

#### 2.1.1. Analysis of the Ultrasound-Assisted Extraction (UAE) Model

The efficiency of UAE of phenolic compounds from E. dysenterica fruit by-products was investigated using the Box–Behnken model in two sets of experiments (Model 1 and Model 2), considering three factors: time, ethanol content (EtOH), and plant material to solvent ratio (PSR). The factors were tested at three levels: 10, 20, and 30 min (Model 1) and 20, 40, and 60 min (Model 2); 20, 40, and 60% ethanol content (Models 1 and 2) and PSR of 0.01; 0.1 and 0.19 g/mL (Models 1 and 2). The definition of these ranges of variables was based on previous studies on the extraction of phenolic compounds in species of the family Myrtaceae [35,36,42].

The time range of 20 to 60 min was taken into account in this study. This interval was selected because long extraction times can increase the likelihood of oxidation and polymerization of phenolic compounds [43]. For the ethanol content, the range used was from 20 to 60%. The efficiency of solubilizing phenolic compounds, particularly those with lower hydrophilicity, is lower for very low concentrations of ethanol (<20%). However, concentrations over 60% can cause the precipitation of certain metabolites and unwanted extraction of non-polar compounds, such as lipids and waxes [36,39].

Fifteen experiments were performed for each model, and the concentrations of total phenolic compounds are listed in Table 1 and Table 2.

For Model 1, the coefficient of determination R^2^ was 0.97575, and the adjusted R^2^ was 0.93211. The model was significant (*p* = 0. 0000002), indicating no lack of fit (*p* = 0.262976). Model 2 was also significant (*p* = 0.0000007), indicating no lack of fit (*p* = 0.263143); the coefficient of determination R^2^ was 0.92478, and the adjusted R^2^ was 0.78939.

The ANOVA results for the data from model 1 are listed in Table 3. A positive and significant linear effect of time (1L) and PSR (3L) was found, suggesting that longer times and higher PSR increase the concentration of total phenolic compounds (expressed as tannic acid) in Model 1. The significant quadratic effect observed for ethanol content (2Q) suggests that the optimal levels for this variable lie between the mean values used in the experiments. In addition, the lack of a significant effect from the tested levels of ethanol content (2L) indicates that this factor has little influence on the extraction process (Table 3).

For Model 2 (Table 4), the ANOVA results revealed a significant quadratic effect for the ethanol content (2Q), again suggesting that the optimal levels are situated between the mean values used in the experiments of Model 2 (Table 4). Only EtOH showed a significant quadratic effect on the total phenol concentration, implying that the relationship between ethanol concentration and total phenols does not follow a linear pattern. In this model, extraction time and PSR did not exhibit significant effects on the total phenol concentration for the variables and levels studied.

#### 2.1.2. Response Surface Analysis (RSA)

**Figure 1:**
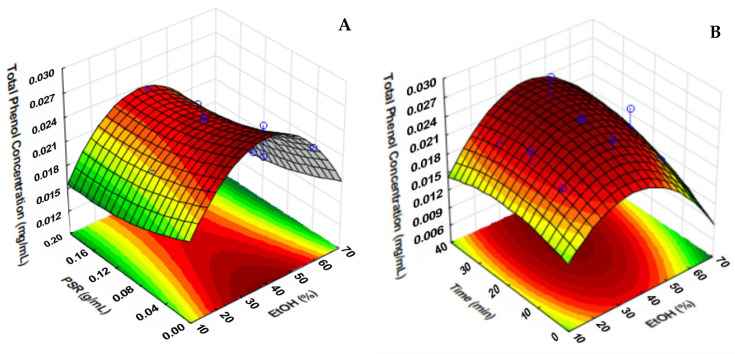
Response surface plots showing the interaction effects of (**A**) PSR (g/mL) and EtOH (%), (**B**) EtOH (%), and time (min) on the extraction of total phenols from *E. dysenterica* fruit by-products (Model 1).

**Figure 2:**
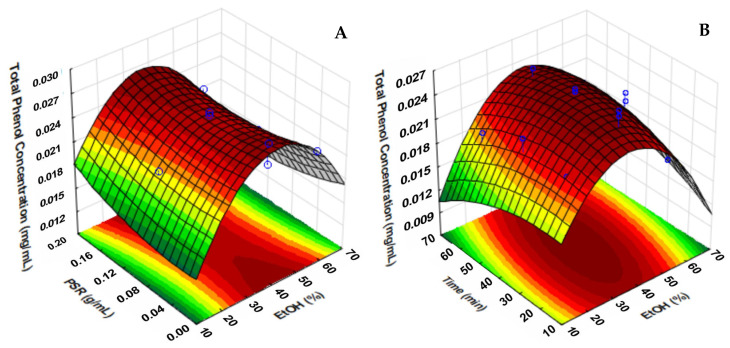
Response surface plots showing the interaction effects of (**A**) PSR (g/mL) and time (min) and (**B**) EtOH (%) and time (min) on the extraction of total phenols from *E. dysenterica* fruit by-products (Model 2).

The response surface plots of Models 1 and 2 are represented in Figure 1 and Figure 2, respectively. The equations of the models are also presented below:

Total Phenol Concentration = 0.00990426689 + 0.000242492294 A + 0.00070591288 B − 0.0319487319 C −0.00000554736679 A^2^ − 0.00000997927833 B^2^ + 0.11980064 C^2^ + 0.00000335070081 A × B − 0.000513247612 A × C − 0.00000598778607 B × C (1)

Total Phenol Concentration = 0.00916450412 − 0.0124988076 A + 0.0000133877945 B + 0.000810512252 C + 0.121246456 A^2^ − 0.00000205246594 B^2^ − 0.0000110212036 C^2^ + 0.000269981533 A × B − 0.000494679461 A × C + 0.00000291998421 B × C (2)

Figure 1A shows that with average EtOH values, there is an increase in the concentration of total phenols extracted in smaller proportions of plant material. In addition, Figure 1B demonstrates that, in addition to the EtOH concentration, the interaction with the extraction time enhances the yield of total phenols, indicating that optimal conditions have EtOH values between 30 and 40% for Model 1. In Figure 2A, the highest and lowest PSR used in the model at ethanol concentrations between 30 and 50% led to the highest concentrations of phenolic compounds. In Figure 2B, the interaction with the extraction time strengthens the importance of the EtOH concentration in the yield of total phenols, indicating optimal values between 45 and 55%, according to ANOVA.

For Model 1, the best extraction conditions of phenolic compounds are PSR = 0.01 g/mL, time = 40 min, and EtOH = 30%. For Model 2, they are PSR = 0.19 g/mL, time = 39 min, and EtOH = 46%.

The optimized extraction conditions are consistent with other studies conducted on Myrtaceae species, such as *Myrcia amazonica* DC. and *Myrtus communis* L., where EtOH and extraction time significantly influenced the yields of phenolic compounds. However, our results show that 46% ethanol content was more effective for *E. dysenterica*, in contrast to the 65% used in *Myrcia amazonica* DC leaf extract and *Myrtus communis* L. pericarp extract [35,36].

The optimization of UAE conditions such as temperature, ethanol content, and time is crucial to maximize extraction yield [36]. The UAE was optimized for the extraction of phenolic compounds in different types of agro-industrial waste, such as chestnut peels and brewer’s bagasse, using factorial designs and surface response methodology [44,45].

The use of UAE-based extraction technologies results in fewer solvents being used and a direct impact on energy, time, and cost expenditures, reducing them [46]. The results of this work are promising for the implementation of these bioinputs on a large scale despite still facing technical and economic challenges [39]. Buvaneshwaran et al. [47] demonstrate that UAE’s application valorizes waste compounds from food processing at an industrial level, as evidenced by extracting phenolic compounds in the grain and coffee processing industry, polyphenols in the wine processing industry, and fruit and vegetables.

#### 2.1.3. Quantitative Analysis of Phenolic Compounds in Extracts Obtained Under Optimized Conditions and in LQE

The concentrations of phenolic compounds predicted by the models under the optimal conditions were 0.028015 mg/mL for Model 1 and 0.026221 mg/mL for Model 2. Using spectrophotometric techniques [34], the mean experimental values of phenolic compounds were 0.030 ± 0.0484 for Model 1 and 0.027 ± 0.005 for Model 2. These values correspond to 107.09% and 102.97% of the concentrations of phenolic compounds predicted by Models 1 and 2. Thus, both models were efficient in the extraction of phenolic compounds since they are capable of extracting values close to and consistent with the values predicted by the models, confirming that both are the best extraction conditions.

Therefore, the choice between using the extraction conditions of Model 1 or Model 2 will depend on the applicability. In Model 1, with a PSR of 0.01 g/mL, a larger amount of solvent would be necessary to extract quantities of phenolic compounds similar to those in Model 2. This represents a high cost with solvents and the concentration of extracts for solvent removal in large-scale extraction processes. On the other hand, in Model 2, with a PSR of 0.19 g/mL, the amount of solvent used will be lower as long as 46% EtOH is used. This difference in the amount of solvent required for extraction makes Model 2 more efficient and viable for large-scale extract production, with lower demand for EtOH and consequent reduction in operating costs, which is why the parameters of this model were selected to obtain the bioinputs in the present work. Model 1 can be used to obtain extracts on a small scale, such as in sample preparation for qualitative and quantitative quality control analysis of plant material.

Extraction temperature and solvent composition were the most influential parameters for UAE in studies carried out with *Morus nigra* pulp [34]. For Myrtaceae, the extraction of phenolic compounds from *Myrtus communis* L. pericarp [35] and *Myrcia amazonica* DC leaves [36] was optimized using the same parameters as in this study, demonstrating that ethanol concentration, UAE time, and liquid–solvent–solid ratio directly affect the concentration of total phenolic compounds in the extract. Similarly, our results confirmed the importance of these parameters, especially EtOH and extraction time, which significantly affected the concentration of total phenols. The optimization of these factors is essential to maximize extraction efficiency and increase the yield of bioactive compounds.

Ultrasound-assisted extraction methods for phenolic compounds are used in different plants and their parts. Extraction methods for phenolic compounds in purple sweet potato [48], in seed shells of *Euryale ferox*, popularly known as prickly waterlily [49], and in leaves of *Cenostigma macrophyllum*, popularly known as “caneleira” [50], led to higher yields of phenolic compounds, when they combined the ultrasound-assisted extraction technique with factorial design and RSM.

#### 2.1.4. Qualitative Analysis of Phenolic Compounds by HPLC-DAD in Bioinputs

**Figure 3:**
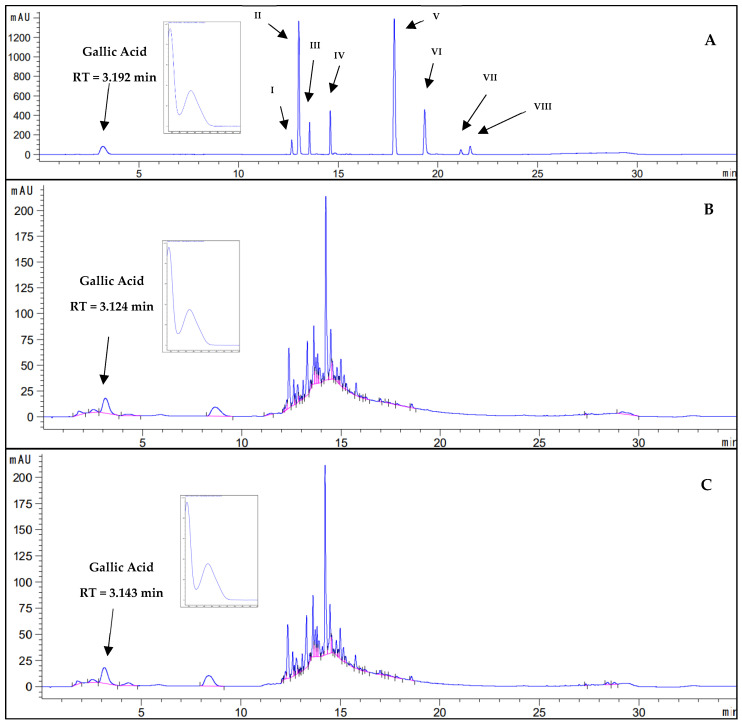
Chromatographic profiles in HPLC-DAD and UV-Vis spectral curves: (**A**) Reference chemical substances. I: catechin, RT (retention time) 12.66 min; II: epicatechin; RT 13.55 min; IV: rutin, RT 14.60 min; V: resveratrol, RT 17.81 min; VI: quercetin, RT 19.37 min; VII: apigenin, RT 21.15 min; VIII: kaempferol, RT 21.61 min; ellagic acid was not detected in this analytical method; (**B**) liquid extract from *E. dysenterica* fruit by-products optimized and concentrated in a rotary evaporator. (**C**) Lyophilized extract from *E. dysenterica* fruit by-products. Chromatographic conditions—C-18 column (4.6 × 100 mm 2.7 µm), mobile phase consisting of acetonitrile and 0.2% aqueous acetic acid solution in gradient (2:98–5 min; 5:95–3 min; 20:80–3 min; 25:75–3 min; 40:60–7 min; 80:20–3 min; 90:10–3 min; 5:95–3 min; 2:98–5 min), flow rate of 1 mL/min, injection volume of 5 µL, column oven temperature of 30 °C, wavelength of 280 nm.

**Figure 4:**
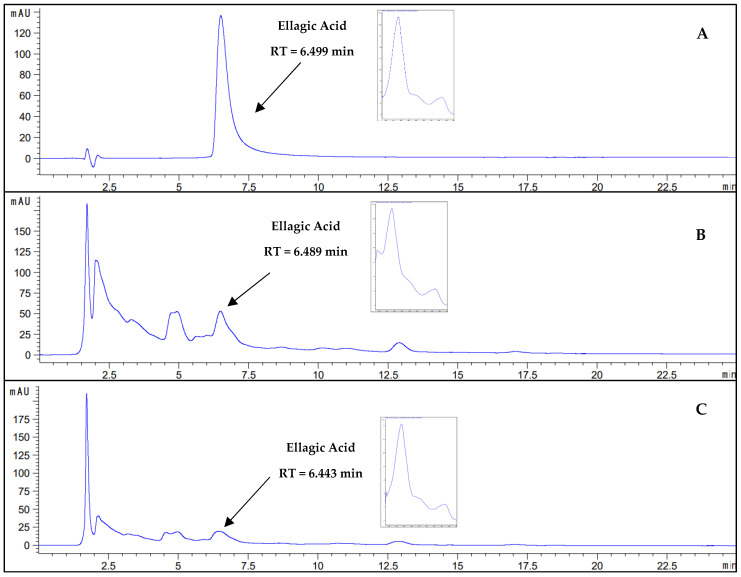
Chromatographic profiles in HPLC-DAD and UV-Vis spectral curves. (**A**) Ellagic acid analytical standard. (**B**) Liquid extract from *E. dysenterica* fruit by-products. (**C**) Lyophilized extract from *E. dysenterica* fruit by-products. Chromatographic conditions—C-18 column (4.6 × 100 mm 2.7 µm), mobile phase consisting of water and acetonitrile (85:15), both acidified with 0.05% (*w*/*v*) trichloroacetic acid, flow rate of 1 mL/min, injection volume of 10 µL, column temperature of 40 °C, wavelength of 254 nm.

The phenolic compounds ellagic acid and gallic acid were confirmed in the bioinputs (LQE and LYE) of *E. dysenterica* by HPLC-DAD, as shown in Figure 3 and Figure 4. Their presence was confirmed by comparing the retention times of the peaks detected in the chromatograms of each extract with the retention times of the peaks of the phenolic compound standards. Additionally, the UV-Vis spectral curves of the peaks from the samples and standards were compared.

Gallic acid contributes significantly to the antioxidant capacity of an extract. This is evident from the high phenolic content and the ability to scavenge free radicals, as seen in the extracts from *Eugenia stipitata* fruit and *Eugenia jambolana* seed [51,52]. The presence of gallic acid in *Eugenia* plants is associated with several therapeutic benefits, including hepatoprotective and antidiabetic effects [52].

Ellagic acid acts as a marker compound in the chemotaxonomic classification of Myrtaceae species, aiding in the differentiation between various genera and species within the family [53]. The extracts from *Eugenia uniflora* leaves and *Eugenia involucrata* fruit and seed contain ellagic acid, which contributes to their antioxidant properties [54,55].

In *E. dysenterica*, ellagic acid and gallic acid are present in hydroalcoholic extract of leaves [56], ethanolic extract of pulp, bark, seed, leaf, and fruit [57], and in fruits at different ripening stages [58].

### 2.2. Antioxidant Activity

The total antioxidant activity of LQE and LYE bioinputs from *Eugenia dysenterica* was evaluated using the ABTS•⁺, FRAP, and DPPH methods. The results are listed in Table 5.

The phenolic compounds in *E. dysenterica* are known for their antioxidant activity [18,20,24], directly dependent on the concentration and composition of phenolic compounds [59], which is consistent with the results of this study. Compared to grape marc from the variety Egiodola (21.206 μM Trolox/100 g), LQE and LYE extracts had higher antioxidant activity with the ABTS method [60]. Despite the results of the statistical analysis showing a significant difference in antioxidant activity using the ABTS method, it is evident that both LQE and LYE have antioxidant activity that ensures the presence of phenolic compounds in the LYE bioinput, even after lyophilization.

Lyophilization preserves the bioactive compounds of the extract, ensuring the maintenance of its antioxidant capacity [61,62,63]. This type of analysis enables the evaluation of the general antioxidant potential of bioactive molecules in the bioinput [64].

### 2.3. Antimicrobial and Antibiofilm Activity

MIC and MBC were determined using the microdilution method. The results are presented in Table 6.

The antimicrobial activity evaluation of the LQE and LYE bioinputs from *E. dysenterica* revealed varying values of MICs and MBCs for the microorganisms tested. In general, both bioinputs inhibited the growth of pathogenic microorganisms, with special relevance for clinical isolates of bovine mastitis and commercial strains.

The lowest MIC and MBC of LQE and LYE (2.5 mg/mL) were found for *Pseudomonas aeruginosa*, isolated from clinical cases of bovine mastitis, indicating sensitivity to the bioactive compounds in the extracts. On the other hand, *Proteus vulgaris* was more resistant, with high MBC values for LQE (>500 mg/mL). A difference in response was also observed between Gram+ bacteria (*Staphylococcus* spp., *Streptococcus* spp., and *S. coagulase* (-)) and Gram- (*Proteus* spp., *Klebsiella* spp., *Escherichia coli*, *Pseudomonas aeruginosa*, and *Proteus vulgaris*), with Gram+ bacteria generally showing greater sensitivity to the extracts tested. These results suggest that the *E. dysenterica* LQE and LYE bioinputs have promising antimicrobial potential, especially against more susceptible microorganisms.

Bovine mastitis, often caused by microorganisms such as *Staphylococcus* spp., *Streptococcus* spp., and *Escherichia coli*, can significantly damage the health of dairy cows and the quality of milk produced [65]. The *Staphylococcus* and *Streptococcus* strains, with MICs of 10 mg/mL and 5 mg/mL and MBCs of 25 mg/mL and 50 mg/mL for LQE and LYE, respectively, indicate that the bioinputs have relevant activity against the main microorganisms responsible for intramammary infections in cattle. *Staphylococcus* infections are particularly challenging, given their ability to form biofilms, which makes treatment more difficult and increases the risk of resistance to traditional antibiotics [66].

*Pseudomonas aeruginosa* is a pathogen known for its resistance to multiple antibiotics and is present in respiratory infections and wounds, both in humans and animals [67]. The antimicrobial activity against *P. aeruginosa* in broiler chicken liver samples, with a MIC of 2.5 mg/mL, suggests that bioinputs have the potential to control this bacterium in veterinary settings. Similar findings were evidenced for the ethanol extracts of *Punica granatum* peels and *Syzygium aromaticum* flowers, which showed MIC ranging from 2.5 to 5.0 mg/mL for *P. aeruginosa* and *Streptococcus* spp. [68].

To evaluate the antibiofilm activity, samples were analyzed for biofilm production based on their optical density (OD) and classified as non-producers, weak producers, moderate producers, and strong producers of biofilm [69] for LQE (Table 7) and LYE (Table 8).

Biofilm formation is advantageous for mastitis-causing pathogens, as it facilitates their persistence in the udder. This ability to form biofilms is associated with recurrent mastitis infections, contributing to increased resistance to antimicrobials and evasion of the host’s immune system [70].

According to Table 7 and Table 8, biofilm production was reduced when LQE and LYQ concentrations were higher, which suggests a direct correlation between bioinputs concentration and inhibition of biofilm production.

Considering the Gram + bacteria group, LQE (Table 7) showed total inhibition of biofilm formation for *S. coagulase* (50 mg/mL) and *Streptococcus* spp. (12.5 mg/mL) and weak biofilm production for *Staphylococcus* spp. (12.5 mg/mL). For Gram-bacteria, LQE showed complete inhibition of biofilm formation for *P. aeruginosa* (liver) (50 mg/mL), *E. coli* (liver) (25 mg/mL), and *P. aeruginosa* (mastitis) (12.5 mg/mL), and weak biofilm production for *Klebsiella* spp. and *E. coli* (heart and mastitis) (12.5 mg/mL), and *P. aeruginosa*–ATCC (25 mg/mL). The extract did not inhibit biofilm production in *Proteus* spp. and *E. coli* (ATCC).

As shown in Table 8, the concentrations tested for LYE were higher than LQE due to the characteristics of each extract. Regarding the group of Gram+ bacteria, LYE showed complete inhibition of biofilm formation for *S. coagulase* (125 mg/mL), *Streptococcus* spp. (31.2 mg/mL), and *Staphylococcus* spp. (125 mg/mL). For Gram-bacteria, LYE showed complete inhibition of biofilm formation for *Proteus* spp. (500 mg/mL), *Klebsiella* spp. (250 mg/mL), *P. aeruginosa* (liver) (62.5 mg/mL), *E. coli* (mastitis) (500 mg/mL), and *P. aeruginosa* (mastitis) (62.5 mg/mL), and weak biofilm production for *E. coli* (heart and liver) (31.2–15.6 mg/mL), and *P. aeruginosa*–ATCC (15.6 mg/mL), and *E. coli* (ATCC) (125 mg/mL).

There are no studies in the literature on antimicrobial activity for extracts from *E. dysenterica* fruit by-products. However, the effect of the ethanolic extract from *E. dysenterica* leaves against *S. aureus* (MIC: 2 mg/mL) and *P. mirabilis* (MBC: 1 mg/mL) is evidenced [71], as is the action of the aqueous extract of the leaves against *S. aureus* strains isolated from human wounds, *S. aureus* (ATCC 12692 and ATCC 29737) (MIC: 83 μg/mL), and against β-lactamase positive *S. aureus* strains, *S. aureus* isolated from human wounds, *S. aureus* enterotoxin positive, and *S. aureus* (ATCC 25904) (167 μg/mL) [11]. For the ethanolic extract of *E. dysenterica* pulp encapsulated with gum arabic or inulin, the inhibitory activity against *S. aureus* and *Listeria monocytogenes* (MIC = 1.48 mg/mL; 0.48 mg/mL) stands out [12]. The essential oil from *E. dysenterica* leaves also has antimicrobial action against *Streptococcus mitis* (MIC = 250 μg/mL), *S. sanguinis* (MIC = 200 μg/mL), *S. sobrinus* (MIC = 400 μg/mL), *S. salivarius* (MIC = 400 μg/mL), and *S. mutans* (MIC = 31.2 μg/mL) [12].

The use of bioinputs as alternatives for the control of these microorganisms can be an important strategy, reducing the dependence on synthetic antibiotics in livestock farming. Bioinputs from *E. dysenterica* present antimicrobial and antibiofilm potential, according to our findings.

## 3. Materials and Methods

### 3.1. Plant Material

*Eugenia dysenterica* fruit was collected in the municipality of Rianápolis, state of Goiás, Brazil (15°29′18′′ S, 49°27′57′′ W), belonging to the São Patrício Valley region (SISGEN Registration A1D543E), in areas with typical Cerrado vegetation. The studied material was identified and registered in the Herbarium of the State University of Goiás–Henrique Santillo Campus with registration number 15137.

After collection, the fruit was selected according to color, ripeness, texture, and absence of injuries and washed in running water. Then, the fruits were depulped in an industrial depulper (Bonina, Itabuna, Brazil) to separate the pulp from the peel and seed. The by-products (peels and seeds) were dried in a forced air oven (Marconi, model MA035/5, Piracicaba, Brazil) at 40 °C until moisture was between 8 and 14% [72]. Afterward, the dried material was pulverized in a knife mill (SP Labor, model SP-33, Presidente Prudente, Brazil) and stored in a sealed plastic bag protected from light.

### 3.2. Physical and Chemical Characterization of the Plant Material

The pharmacognostic characterization [60] of the *E. dysenterica* by-products revealed a moisture content of 8.53% ± 0.23, total ash of 1.36% ± 0.16, swelling index of 0.5 mL, and powder particle size of 1.70 mm (0.19%), 710 µm (29.59%), 355 µm (33.67%), 250 µm (10.17%), 180 µm (6.83%), and 125 µm (19.55%), which classifies it as coarse powder. Phytochemical screening [73,74,75] revealed tannins, anthraquinone heterosides, and phenolic compounds in the plant material.

### 3.3. Optimization of Ultrasound-Assisted Extraction

To optimize the ultrasound-assisted extraction (UAE) of total phenolic compounds, the factors ethanol content (% w/w) of the extractor liquid, extraction time (min), and plant material/solvent ratio (PSR-g/mL) were investigated in two sets of experiments (Model 1 and Model 2) using a Box–Behnken model and the response surface methodology (RSM). For this purpose, 15 random experiments were generated for each model using the Statistica^®^ software, version 12.0. The factors were tested at three levels: 10, 20, and 30 min/20, 40, and 60 min; 20, 40, and 60% ethanol content and PSR of 0.01; 0.1, and 0.19 g/m, in an ultrasonic bath equipment (Eco-Sonics, model Q 5.9/40 A, Indaiatuba, Brazil) 40 kHz, with temperature control of 59 °C [42]. The RSM indicated the best conditions for extracting total phenolic compounds from the plant material. The optimal conditions suggested by the model for both sets of experiments were experimentally validated in triplicate.

### 3.4. Total Phenol Determination

The concentration of total phenols in all samples was determined using a spectrophotometric method [76]. Briefly, for the calibration curve, the reaction standard was prepared using 100 mg of tannic acid for 100 mL of distilled water, and the calibration curve was performed following the respective dilutions, 0.050, 0.075, 0.100, 0.125, and 0.150 mg/mL, and for the extracts, dilutions of 0.050 mg/mL were used for Model 1 and 0.300 mg/mL for Model 2 and 0.02 mg/mL for LQE. One mL of each preparation was poured into tubes containing 2 mL of sodium lauryl sulfate/triethanolamine solution and 1 mL of ferric chloride solution (0.162% FeCL_3_). The reading of the samples and tannic acid concentrations were made after 15 min in a spectrophotometer (Metash, model ESPEC UV-5100, Shangai, China) at 510 nm. The blank consisted of 1 mL of distilled water, 2 mL of sodium lauryl sulfate/triethanolamine, and 1 mL of the ferric chloride solution. To calculate the concentration of total phenols in the analyzed samples, the following equations were used:

(3) $$C=\frac{Absorbance-A}{B}$$

where

C = tannic acid concentration in mg/mL;

A = linear coefficient of the line equation (linear regression of the tannic acid calibration curve);

B = angular coefficient of the line equation (linear regression of the tannic acid calibration curve).

(4) TP=C∗DF

where

TP = Total Phenols (mg/mL);

C = tannic acid concentration in mg/mL;

DF = dilution factors of samples and plant materials to express the concentration of total phenols in the plant material.

### 3.5. Preparation of LQE and LYE Bioinputs

The LQE of *E. dysenterica* fruit by-products was produced according to the optimal conditions obtained in Model 2 of the optimization. For this purpose, we used 1.3 kg of plant material and 6.84 L of EtOH (46%) for 39 min at 59 °C in UAE (Ultronique Q5.9/40A, frequency 40 kHz and power 200 W, Indaiatuba, Brazil). The extract was filtered and concentrated in a rotary evaporator (Ika 10, Staufen, Switzerland) at 40 °C, 25 rpm rotation, and 70 mBar pressure, until approximately 5 L of LQE was obtained. This was homogenized and stored in a plastic bottle at −20 °C. A portion of the liquid extract was lyophilized (Liobras K108, São Carlos, Brazil) in the Bioassay Laboratory of the State University of Goiás to obtain the LYE and reserved for later tests.

### 3.6. Physical and Chemical Characterizations of LQE and LYE Bioinputs

The LQE bioinput presented solids content of 8.64% ± 0.06, pH of 4.21 ± 0.02, relative density (g/mL) of 1.0063 ± 0.0022, ethanol content of 0% (1.0127 g/cm^3^) ± 0.0134 [72], and total phenol concentration of 0.063 mg/mL [76]. The LYE presented a yield of 69.72% and a loss by desiccation of 8.12% ± 0.08.

### 3.7. Qualitative Analysis of Phenolic Compounds in Bioinputs by HPLC-DAD

High-Performance Liquid Chromatography with Diode Array Detector (HPLC-DAD) analysis was performed on an Agilent Technologies 1260 Infinity II HPLC equipment (Santa Clara, United States), OpenLab CDS software (B.01.03.092 version), equipped with a diode array detector (DAD model G7115A), automatic injector, and C18 column (4.6 × 100 mm 2.7 µm) to investigate the occurrence of catechin, epicatechin, caffeic acid, kampferol, gallic acid, ellagic acid, quercetin, rutin, apigenin, and resveratrol in LQE and LYE samples. Two chromatographic conditions were investigated: A) Mobile phase consisting of acetonitrile and 0.2% aqueous acetic acid solution in gradient (2:98–5 min; 5:95–3 min; 20:80–3 min; 25:75–3 min; 40:60–7 min; 80:20–3 min; 90:10–3 min; 5:95–3 min; 2:98–5 min), with a flow rate of 1 mL/min, injection volume of 5 µL, column oven temperature of 30 °C, at wavelengths of 280, 306 and 340 nm [77]. B) Mobile phase consisting of water and acetonitrile (85:15), both acidified with 0.05% (*w*/*v*) trichloroacetic acid, with a flow rate of 1 mL/min, injection volume of 10 µL, column temperature of 40 °C, at wavelengths of 254 nm and 280 nm, for 25 min [64].

### 3.8. Antioxidant Activity

#### 3.8.1. ABTS

The total antioxidant activity was determined by the 2,2′-azinobis (3-ethylbenzothiazoline-6-sulfonic acid) radical (ABTS+) method according to Rufino et al. [78], with modifications. From concentrations of 4000 mg/L of LQE and LYE, 6 dilutions were made, obtaining the following concentrations: 2000, 1000, 500, 250, 128, and 64 mg/L. An aliquot of 30 μL of each dilution was transferred to test tubes, in duplicate, and added with 3 mL of the ABTS-+ radical. The tubes were homogenized, and the reading was performed in a spectrophotometer (Metash, model ESPEC UV-5100, Shangai, China) after 6 min at 734 nm. The blank was composed of ethyl alcohol, and the standard curve was made using Trolox at concentrations of 100 μM, 500 μM, 1000 μM, 1500 μM, and 2000 μM. The procedure was performed in duplicate in a dark environment. The results were expressed in μM Trolox equivalents/g extract. 

#### 3.8.2. FRAP

The determination of total antioxidant activity by the iron reduction method (FRAP) was carried out according to the modified method of Rufino et al. [79]. From concentrations of 4000 mg/L of LQE and LYE, 6 dilutions were made, obtaining the following concentrations: 2000, 1000, 500, 250, 128, and 64 mg/L. An aliquot of 90 μL of each dilution was transferred to test tubes, in duplicate, containing 270 μL of distilled water and 2.7 mL of the FRAP reagent. The tubes were homogenized and kept at rest for 30 min, protected from light. The blank consisted of the FRAP reagent, and the readings were taken at 595 nm using a spectrophotometer (Metash, model ESPEC UV-5100, Shangai, China). The standard curve was constructed using ferrous sulfate at concentrations of 500 μM, 1000 μM, 1500 μM, and 2000 μM. The standard curve was performed in duplicate in a dark environment. The results were expressed in μM FeSO_4_/g sample.

#### 3.8.3. DPPH

The total antioxidant activity was determined by the 2,2-diphenyl-1-picrylhydrazyl (DPPH) radical method according to the modified method of Rufino et al. [80]. From concentrations of 4000 mg/L of LQE and LYE, 6 dilutions were made, obtaining the following concentrations: 2000, 1000, 500, 250, 128, and 64 mg/L. After the kinetic test, which defines the absorbance stabilization of the different concentrations, a time of 22 min was set for reading the LQE and LYE samples. Thus, an aliquot of 100 μL of each dilution was transferred to test tubes, in duplicate, to which 3.9 mL of the DPPH radical was added. The tubes were homogenized and read after 22 min at 515 nm. The blank was composed of methyl alcohol, and the standard curve was performed using the DPPH solution in concentrations of 10 μM, 20 μM, 30 μM, 40 μM, 50 μM, and 60 μM. The procedure was performed in duplicate in a dark environment.

The antioxidant production in the different methods was compared using Dunn’s post hoc test with 95% significance.

### 3.9. Antimicrobial Activity

#### 3.9.1. Minimum Inhibitory Concentration and Minimum Bactericidal Concentration

The antimicrobial activity of LQE and LYE was investigated by the broth microdilution test, according to the protocol recommended by the Clinical and Laboratory Standard Institute (CLSI) for antimicrobial susceptibility testing by dilution of antimicrobial agents in broth [81].

Strains isolated from milk of cows with clinical and subclinical mastitis in different regions of the State of Goiás and donated by the Microbiology Laboratory of the School of Veterinary Medicine and Animal Science of the Federal University of Goiás (UFG) (*Staphylococcus* spp.; *Streptococcus* spp.; *Escherichia coli*) were used. Strains isolated from livers (*Pseudomonas aeruginosa*; *Staphylococcus coagulase (-)*; *Proteus vulgaris*; *Escherichia coli*) and hearts of broiler chickens (*Proteus* spp.; *Klebsiella* spp.; *Escherichia coli*) from commercial slaughterhouses under the state inspection service of the State of Goiás (Agrodefesa) and two standard American Type Culture Collection (ATCC) strains (*Pseudomonas aeruginosa* ATCC 27855; *Escherichia coli* ATCC 25312) maintained at the Microbiology Laboratory of the State University of Goiás (UEG) were also used.

For the samples, a solution was prepared, which contained 5% DMSO, Mueller Hinton (MH) broth, and the mass of LQE and LYE corresponding to concentrations of 50 and 500 mg/mL, respectively. From the initial solution, the samples were diluted for analysis. For LQE, the following concentrations were tested: 50, 25, 12.5, 6.25, 3.13, and 1.5 mg/mL. For LYE, the concentrations were 500, 250, 125, 62.5, 31.2, 15.6, 7.8, 3.9, and 1.9 mg/mL. In sterile 96-well flat-bottom microplates with lids, 50 μL of Mueller Hinton (MH) broth adjusted with bacterial inoculum at 1 × 10^6^ CFU.mL^−1^ and 50 μL of bioinput diluted in MH were placed. As a growth control, a suspension of microorganisms in a medium without extracts was used. To control antimicrobial activity, chloramphenicol was used at concentrations of 64, 32, 16, 8, 4, and 2 μg. mL^−1^. A control was also carried out with hypochlorite (2.5%), a disinfectant used in milking environments, and broth sterility control.

The bacterial inocula were prepared from a suspension of colonies grown in MH agar for 24 h. Colonies were transferred to sterile MH broth until a turbidity of 0.5 on the McFarland scale was reached, resulting in an inoculum concentration of 10^6^ CFU.mL^−1^. After incubating the microplate in an oven for 24 h, 25 µL of freshly prepared 0.01% resazurin was added to each well and incubated at 37 °C for one hour. The presence of bacterial growth was indicated by a pink color, while a blue color indicated the absence of growth. The contents of the wells that appeared blue were transferred to a Petri dish with MH Agar and incubated at 37 °C for 24 h to evaluate the bactericidal or bacteriostatic activity. The Dunn’s post hoc test with 95% significance was used between doses for each group of bacteria to compare different microbial activities.

#### 3.9.2. Effect of LQE and LYE on Biofilm Formation

The quantification and inhibition of biofilm production in polystyrene microtiter plates were performed according to the protocol established by Stepanovic et al. [69] using the same microorganisms as in the previous tests. To form and quantify the total biofilm mass, three to four isolated colonies were suspended in a tube containing 4.5 mL of sterile saline solution. The turbidity of this suspension was adjusted to match the 0.5 McFarland scale, which corresponds to approximately 1.5 × 10^8^ CFU/mL. This inoculum (100 μL) was transferred to a tube containing 9.9 mL of Tryptone Soy Broth (TSB) to adjust the inoculum to a concentration of approximately 1.5 × 10^6^ CFU/mL.

For the biofilm inhibition test, we used 50 μL of the inoculum and 50 μL of LQE and LYE prepared with 5% DMSO and TSB broth corresponding to concentrations of 50 and 500 mg/mL, respectively. The samples were diluted for analysis from the initial solution. For LQE, the following concentrations were tested: 50, 25, 12.5, 6.25, 3.13, and 1.5 mg/mL. For LYE, the concentrations were 500, 250, 125, 62.5, 31.2, 15.6, 7.8, 3.9, and 1.9 mg/mL. The plates were incubated at 35 °C for 24 h to allow the development of microbial biofilms.

The culture media containing the bacterial growths were removed from the wells and washed three times with 150 µL of sterile saline solution to remove non-adherent cells. Each well was added with 110 µL of 1% crystal violet and incubated in bacteriological incubators at 35 °C for 10 min. The dye was discarded, and the plate was washed five times with 150 µL of sterile saline solution.

The plates were dried at 35 °C for 20 min. Subsequently, 110 µL of absolute ethanol was added to each well to solubilize the staining of the adhered bacteria, and the plate was incubated for an additional 10 min. To read the optical densities (OD) of the microplate wells, an automated reader (Epoch) was used at 492 nm.

The tested strains were classified according to biofilm production levels [69]. Based on the relationship between the optical density (OD) and the optical density of the negative control (ODc), the strains were classified as non-producers, weak producers, moderate producers or strong producers, according to the following formula: OD ≤ ODc = non-producer; ODc < OD ≤ (2XODc) = weak producer; (2XODc) < OD ≤ (4XODc) = moderate producer, and (4XODc) ≤ OD = strong producer.

## 4. Conclusions

The results of our study indicate that the LQE and LYE bioinputs from *E. dysenterica* fruit by-products, obtained by UAE under optimized conditions for phenolic compounds, present antioxidant, antimicrobial, and antibiofilm activity, with promising results against microorganisms of relevance to public and veterinary health, related to bovine mastitis and strains isolated from commercial broiler chicken organs.

The antimicrobial activity against strains of *Escherichia coli*, *Pseudomonas aeruginosa*, *Staphylococcus* spp., and *Streptococcus* spp. suggests that these natural extracts are promising for the development of future preparations that may assist in the controlling of these microorganisms.

The research reinforces the importance of exploring natural compounds for the development of new approaches to disease control, especially in a scenario of increasing bacterial resistance. Nevertheless, additional studies are required to assess the efficacy of bioinputs from *Eugenia dysenterica* fruit by-products under clinical conditions, including safety trials, interactions with conventional treatments, and in vivo and ex vivo studies.

Finally, this study contributes to the growing need for sustainable alternatives in the management of resistant pathogens, pointing to a new direction in the development of control strategies based on natural resources and the use of by-products of these resources, with benefits for both animal health and public health and the environment.

## Figures and Tables

**Table 1:** Box–Behnken experimental design for optimization of ultrasound-assisted extraction (UAE) of total phenolic compounds, expressed as tannic acid (mg/mL), from *Eugenia dysenterica* fruit by-products—Model 1.

EtOH (%)	PSR (g/mL)	Time (min)	Total Phenol Concentration (mg/mL)
40	0.19	30	0.024
20	0.01	20	0.023
20	0.19	20	0.020
40	0.01	10	0.025
40	0.19	10	0.022
60	0.01	20	0.023
20	0.1	10	0.021
60	0.1	10	0.018
60	0.19	20	0.020
40	0.01	30	0.028
40	0.1	20	0.025
20	0.1	30	0.021
40	0.1	20	0.024
40	0.1	20	0.024
60	0.1	30	0.020

EtOH (%): ethanol; PSR: plant material to solvent ratio.

**Table 2:** Box–Behnken experimental design for optimization of ultrasound-assisted extraction (UAE) of total phenolic compounds, expressed as tannic acid (mg/mL), from *Eugenia dysenterica* fruit by-products—Model 2.

EtOH (%)	PSR (g/mL)	Time (min)	Total Phenol Concentration (mg/mL)
20	0.1	60	0.017
40	0.1	40	0.024
60	0.19	40	0.025
60	0.1	60	0.021
40	0.1	40	0.025
40	0.01	60	0.023
60	0.1	20	0.020
40	0.1	40	0.026
20	0.1	20	0.021
60	0.01	40	0.024
20	0.19	40	0.026
40	0.01	20	0.020
40	0.19	20	0.022
40	0.19	60	0.021
20	0.01	40	0.026

EtOH (%): ethanol; PSR: plant material to solvent ratio.

**Table 3:** Analysis of variance (ANOVA) of the quadratic polynomial regression model for the ultrasound-assisted extraction (UAE) of total phenolic compounds, expressed as tannic acid (mg/mL), from *Eugenia dysenterica* fruit by-products for Model 1.

Factor	SS	DF	MS	F	*p*
(1) Time (min) (L)	0.000009	1	0.000009	37.0190	0.025966 *
Time (min) (Q)	0.000001	1	0.000001	4.9272	0.156625
(2) EtOH (%) (L)	0.000002	1	0.000002	9.3906	0.092026
EtOH (%) (Q)	0.000059	1	0.000059	255.1187	0.003897 *
(3) PSR (g/mL) (L)	0.000022	1	0.000022	96.0990	0.010246 *
PSR (g/mL) (Q)	0.000003	1	0.000003	15.0769	0.060382
1L by 2L	0.000002	1	0.000002	7.7896	0.107978
1L by 3L	0.000001	1	0.000001	3.7011	0.194278
2L by 3L	0.000000	1	0.000000	0.0020	0.968275
Lack of Fit	0.000002	3	0.000001	2.9552	0.262976
Pure Error	0.000000	2	0.000000		
Total SS	0.000103	14			

EtOH (%): ethanol; PSR: plant material to solvent ratio; SS: Sum of Squares; DF: Degree of Freedom; MS: Mean Squares; *: significant effects (*p* < 0.05).

**Table 4:** Analysis of variance (ANOVA) of the quadratic polynomial regression model for the ultrasound-assisted extraction (UAE) of total phenolic compounds, expressed as tannic acid (mg/mL), from *Eugenia dysenterica* fruit by-products for Model 2.

Factor	SS	DF	MS	F	*p*
(1) Time (min) (L)	0.000000	1	0.000000	0.2340	0.676346
Time (min) (Q)	0.000002	1	0.000002	3.7016	0.194257
(2) EtOH (%) (L)	0.000000	1	0.000000	0.0706	0.815291
EtOH (%) (Q)	0.000072	1	0.000072	106.7325	0.009240 *
(3) PSR (g/mL) (L)	0.000000	1	0.000000	0.7356	0.481454
PSR (g/mL) (Q)	0.000004	1	0.000004	5.2970	0.147994
1L by 2L	0.000005	1	0.000005	8.1164	0.104288
1L by 3L	0.000001	1	0.000001	1.4051	0.357631
2L by 3L	0.000003	1	0.000003	4.7171	0.161995
Lack of Fit	0.000006	3	0.000002	2.9528	0.263143
Pure Error	0.000001	2	0.000001		
Total SS	0.000097	14			

EtOH (%): ethanol; PSR: plant material to solvent ratio; SS: Sum of Squares; DF: Degree of Freedom; MS: Mean Squares; *: significant effects (*p* < 0.05).

**Table 5:** Total antioxidant activity of LQE and LYE bioinputs from *Eugenia dysenterica* evaluated by the ABTS•⁺, FRAP, and DPPH methods. Different letters mean statistical difference by Dunn post hoc test with 95% of significance.

Bioinput	FRAP μM FeSO_4_/g Sample	ABTS•⁺ μM Trolox/g Sample	DPPH g Sample/g DPPH
LQE	1633.60 a	1633.13 a	73.35 a
LYE	1692.09 a	1379.75 b	83.35 a

Caption: ABTS•⁺: 2,2′-azino-bis(3-ethylbenzothiazoline-6-sulfonic) radical cation; FRAP: iron reduction potential; DPPH: 2,2-diphenyl-1-picrylhydrazyl free radical; LQE: liquid extract; LYE: lyophilized extract.

**Table 6:** Values of minimum inhibitory concentrations (MIC) and minimum bactericidal concentrations (MBC) of the LQE and LYE bioinputs from *Eugenia dysenterica*.

Microorganisms		Bioinputs			
		LQE		LYE	
		MIC (mg/mL)	MBC (mg/mL)	MIC (mg/mL)	MBC (mg/mL)
Broiler chicken heart isolates	*Proteus* spp.	25	>50	62.	>500
	*Klebsiela* spp.	12.5	>50	15.6	125
	*Escherichia coli*	12.5	>50	15.6	>500
Broiler chicken liver isolates	*Pseudomonas aeruginosa*	25	>50	15.6	>500
	*Staphylococcus coagulase (-)*	12.5	>50	15.6	250
	*Proteus vulgaris*	25	>50	125	>500
	*Escherichia coli*	25	>50	25	>500
Clinical isolates of bovine mastitis	*Staphylococcus* spp.	10	>50	10	125
	*Streptococcus* spp.	5	25	5	50
	*Escherichia coli*	5	>50	5	>500
	*Pseudomonas aeruginosa*	2.5	2.5	2.5	2.5
Commercial strains (ATCC)	*Pseudomonas aeruginosa*	2.5	50	2.5	50
	*Escherichia coli*	3.125	50	3.125	50

LQE—liquid extract; LYE—lyophilized extract; MIC—minimum inhibitory concentration; MBC—minimum bactericidal concentration; ATCC—American Type Culture Collection.

**Table 7:** Mean values (±standard deviation) of optical densities of the negative control and samples treated with different concentrations of *E. dysenterica* LQE. * Different letters mean the statistical difference between concentrations by Dunn’s post hoc test with 95% significance.

Microorganisms		OD-LQE (mg/mL)						OD-NC
		50	25	12.5	6.25	3.13	1.5	
Broiler chicken heart isolates	*Proteus* spp.	0.0909 ± 0.008 a	0.0863 ± 0.003 a	0.1053 ± 0.004 ab	0.2240 ± 0.063 b	0.2929 ± 0.050 b	0.3929 ± 0.054 b	0.0408 ± 0.002
	*Klebsiela* spp.	** 0.0784 ± 0.018 a	** 0.0833 ± 0.004 a	** 0.0578 ± 0.006 ab	0.1554 ± 0.069 b	0.2204 ± 0.058 bc	0.2456 ± 0.057 c	0.0568 ± 0.002
	*Escherichia coli*	** 0.0599 ± 0.003 a	** 0.0635 ± 0.006 a	** 0.0776 ± 0.003 ab	0.0897 ± 0.002 b	0.0828 ± 0.007 bc	0.1134 ± 0.002 c	0.0410 ± 0.001
Broiler chicken liver isolates	*P. aeruginosa*	* 0.0385 ± 0.007 a	* 0.0340 ± 0.002 a	** 0.0776 ± 0.010 b	0.0928 ± 0.006 b	0.0989 ± 0.003 c	0.2750 ± 0.051 c	0.0397 ± 0.0002
	*S. coagulase (-)*	* 0.0397 ± 0.005 a	** 0.0771 ± 0.001 ab	** 0.0438 ± 0.005 a	0.0903 ± 0.008 b	1.2180 ± 0.108 bc	1.6312 ± 0.262 c	0.0412 ± 0.010
	*Escherichia coli*	* 0.0861 ± 0.011 a	* 0.0865 ± 0.003 a	0.1287 ± 0.017 ab	0.1842 ± 0.016 b	0.1833 ± 0.006 b	0.2460 ± 0.058 b	0.0593 ± 0.002
Clinical isolates of bovine mastitis	*Staphylococcus* spp.	** 0.0683 ± 0.002 a	** 0.0711 ± 0.003 a	** 0.0959 ± 0.015 ab	0.3902 ± 0.075 bc	0.3056 ± 0.02 c	1.0193 ± 0.135 c	0.0671 ± 0.002
	*Streptococcus* spp.	* 0.0405 ± 0.002 a	* 0.0387 ± 0.002 a	* 0.0401 ± 0.001 a	** 0.0445 ± 0.005 a	0.0839 ± 0.006 b	0.0761 ± 0.003 b	0.0418 ± 0.002
	*Escherichia coli*	* 0.0405 ± 0.005 a	** 0.0492 ± 0.007 a	** 0.0427 ± 0.003 a	** 0.0419 ± 0.001 a	** 0.0796 ± 0.006 b	0.1001 ± 0.008 b	0.0408 ± 0.002
	*P. aeruginosa*	* 0.0395 ± 0.001 a	* 0.0394 ± 0.004 a	* 0.0381 ± 0.003 a	** 0.0428 ± 0.007 ab	* 0.0386 ± 0.003 a	0.0894± 0.005 b	0.0406 ± 0.002
Commercial strains (ATCC)	*P. aeruginosa*	** 0.0922 ± 0.003 a	** 0.0957 ± 0.003 a	0.1439 ± 0.026 ac	0.1618 ± 0.022 ac	0.1921 ± 0.004 bc	0.1989 ± 0.007 bc	0.05833 ± 0.006
	*Escherichia coli*	0.1137 ± 0.013 a	0.1353 ± 0.016 a	0.1940 ± 0.038 ab	0.2222 ± 0.064 b	0.2682 ± 0.094 bc	0.2994 ± 0.104 c	0.0436 ± 0.001

OD: optical density; NC: negative control; * no biofilm production; ** weak biofilm production.

**Table 8:** Mean values (±standard deviation) of optical densities of the negative control and samples treated with different concentrations of *E. dysenterica* LYE. * Different letters mean the statistical difference between concentrations by Dunn’s post hoc test with 95% significance.

Microorganisms		OD-LYE (mg/mL)									OD-NC
		500	250	125	62.5	31.2	15.6	7.8	3.9	1.9	
Broiler chicken heart isolates	*Proteus* spp.	* 0.0400 ± 0.005 a	** 0.0455 ± 0.003 a	** 0.0488 ± 0.008 a	0.0889 ± 0.002 ab	0.0952 ± 0.001 b	0.1053 ± 0.004 b	0.2983 ± 0.035 b	0.3855 ± 0.032 b	0.4846 ± 0.046 b	0.0408 ± 0.002
	*Klebsiela* spp.	** 0.0630 ± 0.003 a	* 0.0567 ± 0.008 a	** 0.0583 ± 0.006 a	** 0.0689 ± 0.005 ab	** 0.0714 ± 0.010 ab	** 0.0747 ± 0.004 b	** 0.0919 ± 0.008 bc	0.1413 ± 0.022 c	0.2053 ± 0.045 c	0.0568 ± 0.002
	*Escherichia coli*	** 0.0486 ± 0.006 a	** 0.0549 ± 0.006 a	** 0.0584 ± 0.006 ab	** 0.0555 ± 0.005 ab	** 0.0505 ± 0.005 b	0.0993 ± 0.012 bc	0.2059 ± 0.116 cd	0.1233 ± 0.010 cd	0.1676 ± 0.033 d	0.0410 ± 0.001
Broiler chicken liver isolates	*P. aeruginosa*	* 0.0390 ± 0.006 a	** 0.0410 ± 0.001 a	* 0.0379 ± 0.007 a	* 0.0395 ± 0.001 a	** 0.0405 ± 0.003 a	* 0.0377 ± 0.007 a	** 0.0726 ± 0.006 b	0.0966 ± 0.012 b	0.2743 ± 0.038 b	0.0397 ± 0.0002
	*S. coagulase (-)*	** 0.0438 ± 0.007 a	* 0.0406 ± 0.002 c	* 0.0387 ± 0.005 b	** 0.0449 ± 0.005 a	* 0.0400 ± 0.004 b	* 0.0404 ± 0.001 c	0.0861 ± 0.011 a	0.8028 ± 0.627 a	0.7484 ± 0.574 a	0.0412 ± 0.010
	*Escherichia coli*	* 0.0590 ± 0.007 a	** 0.0654 ± 0.005 a	** 0.0619 ± 0.010 a	** 0.0678 ± 0.006 a	** 0.0655 ± 0.006 a	** 0.0728 ± 0.010 ab	0.1448 ± 0.038 b	0.1591 ± 0.034 b	0.2188 ± 0.068 b	0.0593 ± 0.002
Clinical isolates of bovine mastitis	*Staphylococcus* spp.	* 0.0658 ± 0.002 a	** 0.0687 ± 0.004 c	* 0.0633 ± 0.001 b	** 0.0716 ± 0.007 a	** 0.0885 ± 0.006 b	** 0.0955 ± 0.011 c	0.1436 ± 0.007 a	0.3397 ± 0.089 a	0.9852 ± 0.140 a	0.0671 ± 0.002
	*Streptococcus* spp.	** 0.0418 ± 0.001 a	* 0.0408 ± 0.007 a	* 0.0400 ± 0.004 a	* 0.0410 ± 0.003 a	* 0.0412 ± 0.009 a	** 0.0448 ± 0.009 b	* 0.0413 ± 0.007 b	** 0.0797 ± 0.002 c	0.1704 ± 0.019 c	0.0418 ± 0.002
	*Escherichia coli*	* 0.0389 ± 0.010 a	** 0.0415 ± 0.006 a	** 0.0419 ± 0.004 a	** 0.0439 ± 0.010 a	** 0.0519 ± 0.010 ab	** 0.0435 ± 0.009 a	** 0.0423 ± 0.003 a	0.0821 ± 0.005 b	0.0875 ± 0.015 b	0.0408 ± 0.002
	*P. aeruginosa*	** 0.0416 ± 0.004 a	* 0.0386 ± 0.005 a	** 0.0411 ± 0.002 a	* 0.0392 ± 0.001 a	* 0.0390 ± 0.008 a	** 0.0586 ± 0.009 ab	** 0.0618 ± 0.003 b	** 0.0786 ± 0.006 b	0.0868 ± 0.005 b	0.0406 ± 0.002
Commercial strains (ATCC)	*P. aeruginosa*	** 0.0660 ± 0.003 a	** 0.0702 ± 0.006 a	** 0.0814 ± 0.007 ab	** 0.0925 ± 0.004 b	** 0.1086 ± 0.009 bc	** 0.1104 ± 0.008 bc	0.1272 ± 0.010 cd	0.1366 ± 0.011 de	0.1793 ± 0.027 e	0.05833 ± 0.006
	*Escherichia coli*	** 0.0475 ± 0.008 a	** 0.0693 ± 0.004 a	** 0.0701 ± 0.016 a	0.1137 ± 0.013 a	0.2814 ± 0.041 b	0.4507 ± 0.119 b	0.5653 ± 0.048 b	0.6574 ± 0.131 b	0.8361 ± 0.143 b	0.0436 ± 0.001

OD: optical density; NC: negative control; * no biofilm production; ** weak biofilm production.

## Data Availability

The data presented in this study are available upon request to the corresponding authors.
